# Physical activity and psychological adjustment among retirees: a systematic review

**DOI:** 10.1186/s12889-023-15080-5

**Published:** 2023-01-28

**Authors:** Mehdi Sharifi, Davud Nodehi, Behzad Bazgir

**Affiliations:** 1 Exercise Physiology Research Center, Life Style Institute, Baqiyatallah University of Medical Sciences, Tehran, Iran; 2 Behavioral Sciences Research Center, Baqiyatallah University of Medical Sciences, Tehran, Iran

**Keywords:** Exercise, Leisure, Adjustment, Mental Health, Retirement

## Abstract

**Background:**

Health-related behaviors may change after retirement and induce changes in the mental health. This systematic review aimed to investigate the nature of changes in physical activity and leisure activities, as well as the relationship between physical activity, leisure, and psychological adjustment among retirees.

**Methods:**

Search of papers was done in three electronic databases of PubMed, ISI Web of Science, and Scopus without constraints on time, geographical regions, or languages in February 2022. The papers that had examined the relationship between physical activity and adjustment among retirees using observational design (cross-sectional or longitudinal) were included in the present study. To evaluate the methodological quality of cross-sectional studies, Joanna Briggs Institute (JBI) Critical Appraisal Checklist, and for longitudinal studies, Newcastle-Ottawa Scale (NOS) were used.

**Results:**

The search results identified 1458 records. Twenty-six papers were included in this review based on the inclusion and exclusion criteria. The findings of most of these studies indicated a significant positive correlation between physical activity, leisure, and psychological adjustment in retirees. Retirees were mostly engaged in passive leisure activities such as reading, watching TV and movies, and less engaged in physical activities, sport, or physical exercise. General organized assessment of the total physical activity among retirees was not possible.

**Conclusions:**

Based on the study findings, it can be stated that there is a positive correlation between physical activity, leisure, and the retirees’ adjustment. Usage of the same and valid measurement method specific to old age and retirement period can be useful in more precise assessment of physical activity and its association with adjustment among retirees.

**Supplementary Information:**

The online version contains supplementary material available at 10.1186/s12889-023-15080-5.

## Introduction

Retirement is generally defined as abandoning an occupation and not actively seeking a job [1]. Retirement is a significant turning point in people’s lives as well as important social stress which may affect the physical and mental health [2]. Health-related behaviors may change following retirement and induce changes in the mental health [3]. Physical inactivity is the main cause of death and disease, while physical activity is associated with increased life expectancy as well as decreased risk of developing type II diabetes, coronary heart disease, and some cancers [4, 5]. Meanwhile, physical activity and participation in sports activities play a key role in reducing the symptoms of depression and anxiety as well as enhancing the quality of life and wellbeing [6–9].

Retirement has been known as a turning point in determining the physical activity behaviors in old age, and indicates a key transition affecting physical and social activities [10]. Physical activity is a modifiable behavior which can change depending on the conditions, events, and major transitions of life [11, 12]. Thus, the pattern of physical activity varies with changes in living conditions, such as the retirement period. The results of studies dealing with changes of physical activity levels after retirement have been contradictory [8, 13–15]. Such a contradiction in physical activity levels after retirement can be due to socioeconomic status and previous occupations [16–18].

Mental health may be especially sensitive to changes during the retirement period and research in this regard presents different evidence. Some studies have shown that retirement can have a significant relationship with diminished life satisfaction and more psychological distress; however, in other studies no detrimental or positive effect has been reported [2]. Meanwhile, a systematic review study by Barbosa et al. [19] indicated that a number of studies have introduced physical activity as a protective factor for retirement, while Lee and Hung reported that heavy physical activity may be a risk factor [20]. Investigation of the pattern of physical activity as well as its association with mental health and retirement adjustment (RA) has received less attention.

According to van Solinge and Henkens, adjustment is the process of becoming accustomed to retirement-induced changes of life [21]; it is defined depending on how the person assesses their adjustment or adaptation to the retirement conditions as well as its associated changes. While many people enjoy the freedom offered by retirement, approximately 25% experience a decline in adjustment, which leads to adverse psychosocial consequences [22]. The focus of this study was psychological adjustment in retirement, which encompassed criteria of life satisfaction, quality of life, wellbeing, and mental health.

Adjustment to retirement can definitely be the first step towards successful aging [23]. In most developed countries, retirement is often known as a stage of leisure in the person’s life [24]. The change of the role from a routine lifestyle towards a relatively inactive lifestyle is considered a challenge which can trigger development of mental health problems for the retirees [25]. Meanwhile, freedom from the stress of work and having more opportunity for participation in leisure activities may have a positive effect on mental health and adjustment after retirement [26, 27]. Leisure has been found as a protective factor against the changes that occur in the routine life of people; thus, identifying the effect leisure can have on retirees’ adjustment and generally on this stage of life is crucial [28].

Examining previous similar systematic review studies showed that studies on Factors related to adjustment to retirement [19], changes in physical activity and sedentary behavior across the retirement transition according to socioeconomic status [13, 29], determining the status of research evidence about health promotion at retirement [30], the relationship between different types of retirement and changes in health performance [31], the relationship between retirement and depression [32, 33], factors related to well-being in retirement [34], and examining the dimensions of preparation for old age and retirement [35] has been done. As it is known, none of the previous systematic review studies have investigated the relationship between physical activities and leisure with adjustment in retirees. Conducting this systematic review can increase our knowledge of the relationship between the dimensions of physical health and psychological health in retirees. Also, as mentioned above, health-related behaviors may change during retirement; therefore, examining the nature of changes in physical activity and leisure during retirement, in addition to increasing our knowledge, can provide the basis for further research related to this domain.

The growing body of literature about retirement adjustment has rarely dealt with the role of participation in leisure activities during the adjustment process [26]. Assessment of physical activity alongside the leisure activities in retirement adjustment can provide a wider perspective especially about active leisure (such as physical activity and exercises) in comparison to passive leisure (such as reading and watching television). To our knowledge, no systematic review so far has been conducted on the relationship between physical activity, leisure, and psychological adjustment among retirees. Thus, the goals of this systematic review included examining: (1) Whether there is any relationship between physical activity, leisure, and psychological adjustment among retirees? (2) What is the nature of changes in physical activity and leisure activities among retirees? Summarizing and collating the researched results first can elevate levels of evidence beyond our knowledge from single previous studies, and can also be a guide for future research.

## Method

A systematic review was conducted to investigate the nature of changes in physical activity and leisure activities, as well as the relationship between physical activity, leisure, and psychological adjustment among retirees. The present study protocol was performed according to the Preferred Reporting Items for Systematic Reviews and Meta-Analyses (PRISMA) guidelines [36] and registered in PROSPERO (CRD42022316616).

### Search strategy

The search was performed in three electronic databases (*PubMed, ISI Web of Science* and *Scopus*) without time or language constraints up to 21 February 2022. A manual search was also performed in the reference list of similar systematic review studies. However, none of the studies were included in the present study in this way because they either did not meet the inclusion criteria or were excluded due to duplication. The keywords used were as follows:

1. Concept 1 “Physical Activ*” OR exercise OR sport OR recreation OR “leisure activ*” OR “Physical exercise” OR “motor activ*”
2. *Concept 2 Adjustment OR well-being OR “Quality of Life” OR “Mental health” OR Life Satisfaction*
3. Concept 3. Retirement OR retiring OR retire OR retirees OR retired (retir*)

The search was done as extensive in order to ensure sufficient literature coverage. The search strategy was related to retrieving papers that included each of the three above concepts in the “title/abstract” or medical subject headings (MeSH). In the PubMed database, the “title/abstract” or “medical subject headings (MeSH)” strategy, in the Web of Science database, the “Title” and “Abstract” strategies separately, and in the Scopus database, the “Title/Abstract” strategy was used during the search. The precise process of search strategy is available online in the Additional file 1.

### Study selection

The screening and selection of papers was done by two authors independently and any disagreement was resolved through consensus with a third reviewer.

The inclusion criteria were as follows:

dddd

1. Studies that dealt with assessing physical activity or leisure and psychological adjustment (components described in the PICO: Intervention = physical activity or leisure and Outcome = psychological adjustment).
2. The participants included male and female retirees without age restrictions (components described in the PICO: Population: retirees).
3. The studies had been performed with an observational design (cross-sectional or longitudinal).

Exclusion criteria included the following:

1. Studies that selected retirees with specific health conditions (such as retirees with an illness or retirees who had recently undergone surgery). These retirees may have different leisure time and physical activity than normal retirees, or they may not be physically active.
2. Studies that only studied the elderly population, did not focus on choosing retirees as a research sample. The conditions of a retired person in terms of physical activity and psychological adjustment may be different from that of an elderly person.
3. Studies that have investigated different aims from the present study goals and have reached different results.
4. Studies that have examined the specific age range before retirement (such as youth, and middle age). The focus of this study is on people after retirement.
5. Papers that’s its full text has been non-English.
6. Experimental studies and studies with these characteristics (literature review, books, review papers, unpublished manuscripts, and conference abstracts)

### Quality assessment

To evaluate the methodological quality of cross-sectional studies, Joanna Briggs Institute (JBI) Critical Appraisal Checklist was used, while for longitudinal studies, Newcastle-Ottawa Scale (NOS) was employed. Therefore, the risk of bias was evaluated using two scales, JBI and NOS. The risk of bias in each study was done by two authors independently, and any disagreements between assessors were resolved through consensus with a third reviewer. NOS scale has eight items evaluating four dimensions of sample selection, sample representativeness, comparability, and outcome assessment. Apart from the comparability dimension to which at most two stars are assigned, the other dimensions would be assigned at most one star. More stars indicate higher quality [37].

JBI checklist includes eight items of assessing sample selection criteria, subject description, exposure measurement, subject condition measurement, confounding factors identification, confounding factors control, outcome assessment, and statistical analysis. Each one is classified as yes, no, unclear, or not applicable [38]. For both JBI and NOS designs, the papers with score above 7 would be classified as low risk of bias. The papers with score 5–7 indicate moderate risk of bias, and those with score lower than 5 show high risk of bias.

### Data extraction

The main features that were extracted from the reviewed studies included author, year, country, study design, sample size, the age range or mean, gender ratio, exposure variables, outcome variables, measurement tool, main results, and additional results. After extraction, the information was examined by two authors in terms of data accuracy. Due to heterogeneity in applying the measurement tools as well as the results and measurement of the researched variables, it was not possible to perform meta-analysis. Thus, a systematic review was performed.

## Results

**Fig. 1:**
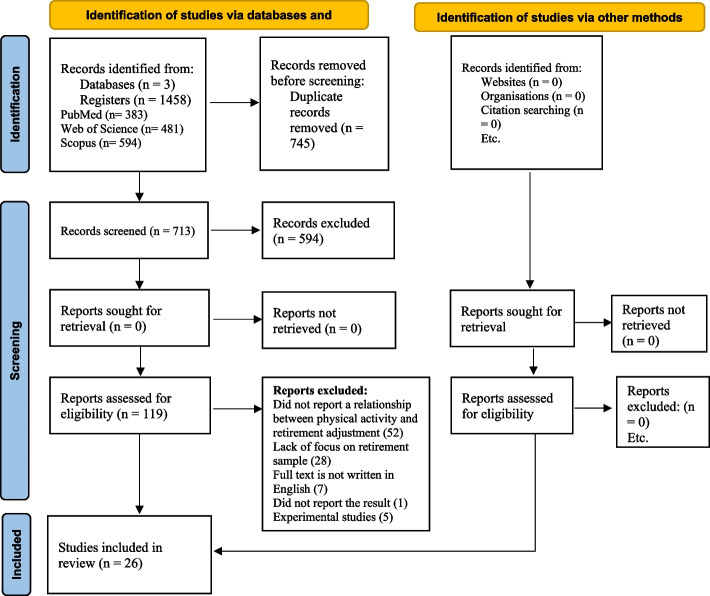
Diagram flow of outcomes of the review

Overall, 1458 papers were identified from the three databases of *PubMed, ISI Web of Science*, *and Scopus*. After removing duplicates and irrelevant cases, as well as abstract analysis resulting from inspecting the title, abstract, and methodology, 119 studies became eligible for investigating the full text. Eventually, 26 papers matched the inclusion criteria and were included in the study. The process of selection of papers has been briefly visualized in PRISMA 2020 flow diagram (Fig. 1).

### Methodological quality

Methodological quality assessments of cross-sectional and longitudinal studies are presented in Tables 1 and 2. For cross-sectional studies, quality assessment scores ranged from 3 (minimum quality) to 8 (highest quality). The quality of longitudinal studies ranged within average (score 5) and good (score 9). From among studies examined in both cross-sectional and longitudinal designs, two studies were classified as high risk of bias [39, 40], three as low risk of bias [41–43], and all other studies as moderate risk of bias (Tables 1 and 2). The study by Fry & Ghosh [39] received score 3 and the one by Read et al. [40] was assigned score 4. The study by Fry & Ghosh had not used a standard instrument with suitable reliability and validity. In the study by Read et al., the exclusion and inclusion criteria had not been presented. Further, in both studies, the standard criteria used for measurement of the condition, strategies to address confounding factors, and identification of confounding factors had not been explained clearly.

**Table 1:** Quality Assessment of Analytical Cross-Sectional Studies Using the Joanna Briggs Institute (JBI) Critical Appraisal Checklist

Source	JBI Critical Appraisal Checklist for Cross-sectional								
	D1	D2	D3	D4	D5	D6	D7	D8	Total Score
Bailey & McLaren 2005 [7]	U	Y	Y	U	U	Y	Y	Y	5
Bevil et al. 1994 [46]	Y	Y	Y	Y	U	Y	Y	Y	7
Brien 1981 [50]	Y	Y	U	Y	U	U	Y	Y	5
Conde-Sala et al. 2017 [44]	U	Y	U	U	Y	Y	Y	Y	5
Earl et al. 2015 [14]	Y	Y	Y	U	U	Y	Y	Y	6
Fly et al. 1981 [54]	Y	Y	U	Y	Y	U	Y	Y	6
Fry & ghosh 1980 [39]	Y	Y	N	U	U	U	N	Y	3
Krahe 2011 [49]	Y	Y	U	Y	Y	Y	U	Y	6
Kuvaja-Köllner et al. 2013 [8]	U	Y	Y	U	U	Y	Y	Y	5
Lee & Hung 2011 [20]	Y	Y	Y	U	U	Y	Y	Y	6
Morgan et al. 1991 [53]	Y	Y	Y	Y	U	U	Y	Y	6
Nimrod & Adoni 2006 [24]	Y	Y	Y	Y	U	U	Y	Y	6
Nimrod 2007 [23]	Y	Y	Y	Y	U	U	Y	Y	6
Nimrod et al. 2008 [52]	Y	Y	U	Y	U	U	Y	Y	5
Oakley & Pratt 1997 [47]	U	Y	Y	U	U	Y	Y	Y	5
Rakhshani et al. 2014 [15]	Y	Y	Y	Y	U	Y	Y	Y	7
Read et al. 2013 [40]	U	Y	Y	U	N	U	Y	Y	4
Reddick & stewart 1994 [48]	Y	Y	U	Y	U	Y	U	Y	5
Russell 1987 [51]	Y	Y	Y	Y	U	U	Y	Y	6
Sener et al. 2007 [43]	Y	Y	Y	Y	Y	Y	Y	Y	8
Walsh et al. 2019 [9]	Y	Y	Y	Y	U	U	Y	Y	6

*Y*  Yes, *U*  Unclear, *N*  NO

D1: Were the criteria for inclusion in the sample clearly defined? D2: Were the study subjects and the setting described in detail? D3: Was the exposure measured in a valid and reliable way? D4: Were objective, standard criteria used for measurement of the condition? D5: Were confounding factors identified? D6: Were strategies to deal with confounding factors stated? D7: Were the outcomes measured in a valid and reliable way? D8: Was appropriate statistical analysis used?

**Table 2:** Quality Assessment of Longitudinal Studies (*n* = 1) Using the Newcastle-Ottawa Scale (NOS)

Source	Criteria								
	Representation of the Exposed Cohort	Selection of the Non Exposed Cohort	Ascertainment of Exposure	Demonstration That Outcome of Interest Was Not Present at Start of Study	Comparability of Cohorts on the Basis of the Design or Analysis	Assessment of Outcome	Follow-Up Was Long Enough for Outcomes to Occur	Adequacy of Follow**-**Up of Cohorts	Total Score
Henning et al. 2021 [26]	★		★	★	★	★	★	★	7
Iwatsubo et al. 1996 [41]	★		★	★	★★	★	★	★	8
Nimrod & Shrira 2016 [45]	★		★	★	★	★	★		6
Olds et al. 2018 [3]			★	★		★	★	★	5
Potocnik & Sonnentag 2013 [42]	★	★	★	★	★★	★	★	★	9

### Study characteristics

Table 3 shows the characteristics of the papers included in the systematic review. There was no constraint regarding year of publication for selection of papers. From among the studies examined, nine had been performed before 1999, five between 2000 and 2009, and 12 from 2010 onwards. All studies had been written in English. Also, 21 studies had been performed as cross-sectional and five as longitudinal. Most studies (*n* = 21) had employed primary data, while 5 had utilized secondary data [8, 26, 42, 44, 45]. Studies had been performed in different countries including Australia (*n* = 6), USA (*n* = 4), Israel (*n* = 2), France, Finland, Taiwan, England, Scotland, Iran, India, Sweden, and Turkey (each country one study), and one study had been done simultaneously in two countries (USA and India), one in two countries of the USA and Israel, and 3 in multiple countries. The continent of Asia and Oceania had the largest contribution in terms of the number of studies conducted (each continent six studies), then 5 studies were conducted in the European continent and 4 studies were conducted in the North American continent. Among the remaining 5 studies, three studies were conducted simultaneously in different countries and continents, and two studies were conducted simultaneously in two continents. Overall, 54,043 retirees had been investigated. The sample size ranged from 32 to 33,241 participants. The participants’ age varied within 50–104 years. Out of all subjects of studies, 53.7% were female and 46.3% were male.

**Table 3:** Main Characteristics of the Included Studies in the Systematic Review

Authors and Year (Country)	Study Design (years of follow up)	Sample size (N) Age (Mean or range) Sex (Male%)	Exposure variables (measurement)	Outcome variables (measurement)	Main Results	Other finding
Bailey & McLaren 2005 [7] (Australia)	CS	194 68 45% M	Physical activity 31-item Yale Physical Activity Survey	Mental health (Depression) Zung Depression Inventory	A significant negative correlation was reported between physical activity performed with others and depression	In the model framework, neither physical activity alone nor with others was able to predict depression or suicidal ideation.
Bevil et al. 1994 [46] (USA)	CS	32 65 to 86 Y 44% M	Leisure Activities Leisure Activities Inventory (LAI) (36-item)	Life satisfaction 18-item Life Satisfaction Index A (LSIA)	Doing more activities and more varied reason for leisure activities were associated with greater life satisfaction.	
Brien 1981 [50] (Australia)	CS	262 - 42% M	Leisure activities (A list of 93 activities was included)	Life satisfaction 10 bipolar items on a 7-point scale.	Regarding overall life satisfaction, the results showed that leisure characteristics were a poor predictor of life satisfaction.	Interaction and Number of leisure activities is directly associated with retirement activity satisfaction, but not with overall life satisfaction.
Conde-Sala et al. 2017 [44] (Multiple)	CS	33,241 74.7 43% M	Physical exercise (Frequency of physical exercise, participation in activities)	Quality of life 12 items originating in the CASP-19	In all countries, physical activity and participation in activities were associated with better quality of life.	
Earl et al. 2015 [14] (Australia)	CS	243 66 48% M	Leisure activities (Activities included social, educational, light and vigorous exercises, chores and home entertainment)	Psychological wellbeing PANAS (Positive and Negative Affect Schedule) short form (Thompson, 2007)	Results showed that some activities but not all were related to better psychological wellbeing. While social activities were associated with higher positive affect, surprisingly light exercise was related to higher negative affect.	Participation in a wider range of activities was associated with higher positive emotions and lower negative emotions.
Fly et al. 1981 [54] (USA)	CS	400 70 50% M	Leisure activity (Converse and Robinson’s (1973) activities list)	Adjustment ladder scale on satisfaction and two questions about happiness and satisfaction	People with more leisure activities were more satisfied with their lives than people with few leisure activities.	
Fry & ghosh 1980 [39] (India & USA)	CS	80 India (50% M) 80 USA (56% M) 65 to 85 Y	Recreation (a single-item question)	Life satisfaction (a single-item question)	The results showed that recreational opportunities for Americans bring more life satisfaction than the Indians.	
Henning et al. 2021 [26] (Sweden)	L (4 years)	1033 64.26 53% M	Leisure activity engagement list of 27 activity (three categories: intellectual, social or physical)	Mental health (depression) 8-item short form CES-D scale	Level and change in intellectual, social or physical leisure activities with depressive symptoms were negatively associated, but the direction of effects was unclear	
Iwatsubo et al. 1996 [41] (France)	L (2 years)	T1 = 627 (51% M) T2 = 464 (47% M) Mean = 63	Leisure activities Three categories: physical, hobby & social activities	Life Satisfaction original version of 20 items Life Satisfaction Index A (LSIA)	Leisure activities at T1 and T2 were significantly related to the LSIA.	
Krahe 2011 [49] (Australia)	CS	116 50 to 91 Y 45% M	Leisure participation use of a 7-day time-use diary recording all activities	Life satisfaction (a single-item question)	The effect of leisure participation on life satisfaction and retirement satisfaction was not significant.	
Kuvaja-Köllner et al. 2013 [8] (Finland)	CS	1,410 55–74 Y 49% M	Physical exercise (duration, frequency and mean intensity of physical exercise during the previous 12 months)	Health-related quality of life (RAND-36)	A higher amount of exercise was associated with physical and mental components of quality of life	
Lee & Hung 2011 [20] (Taiwan)	CS	352 60 Y and above 46% M	Physical exercise Combined assessment of exercise intensity and frequency	Well-being General Well-Being (GWB) schedule - Dupuy	Exercise frequency was positively correlated with well-being, while exercise intensity was negatively correlated with well-being.	Low- to moderate-intensity exercise may be better for older adults’ psychological well-being.
Morgan et al. 1991 [53] (England)	CS	1042 507 aged 65–74; 535 aged 75+ 38% M	Physical activity customary physical activity assessment (CPA)	Psychological well-being - Life Satisfaction Index (LSIZ) - Symptoms of Anxiety and Depression (SAD)	Relationships of physical activity levels with psychological well-being were weak, indirect and gender-specific	
Nimrod & Adoni 2006 [24] (Israel)	CS	383 64.3 42% M	Leisure activities included a list of 41 activities	Life satisfaction Life Satisfaction Index (LSI)	Leisure participation and leisure satisfaction were associated with life satisfaction.	
Nimrod & Shrira 2016 [45] (Multiple)	L (8 years)	7875 71.21 54% M	Leisure activities (Questions about five activities performed in the last month)	Quality of life 12 items originating in the CASP-19	Association between high levels of leisure involvement and quality of life increased with time.	
Nimrod 2007 [23] (Israel)	CS	383 64.3 42% M	Leisure activities a list of activities, and a scale of 11 degrees of participation frequency	Life satisfaction Life Satisfaction Index- ALSI	Leisure even more than many background factors, such as poor health, low income or absence of spouse, contribute to retirees’ life satisfaction.	
Nimrod et al. 2008 [52] (Israel & USA)	CS	USA *N* = 430 66.2 30% M Israel *N* = 383 64.3 42% M	Leisure participation Israel (list of 41 activities) USA (The ACL dataset: three categories of leisure activity: informal, formal, and physical)	Subjective well-being Israel: Life Satisfaction Index (LSI) USA: life satisfaction (through a single item) and depressive symptoms (CES-D) scale	In the Israeli example, increasing the frequency of participation in physical activity was associated with life satisfaction. In the American example, changes in leisure activities had no relationship to life satisfaction.	
Oakley & Pratt 1997 [47] (Scotland)	CS	40 67 37% M	Leisure activity Nottingham Leisure Questionnaire	Life Satisfaction Life Satisfaction Index (LSIA)	A positive correlation was found in relationship between leisure activities and life satisfaction for all subjects.	
Olds et al. 2018 [3] (Australia)	L (2 years)	105 63.4 49% M	Physical activity Multimedia Activity Recall for Children and Adults (MARCA)	Mental health Depression, Anxiety and Stress Scales (DASS21), Short Warwick-Edinburgh Mental Well-being Scale (SWEMWBS)	In retirees, replacing work time with physical activity was associated with better mental health (improvement in depression, anxiety and stress).	
Potocnik & Sonnentag 2013 [42] (Multiple)	L (2 years)	2813 69.79 56% M	Participants in sport Engaging in multiple activities	Quality of life CASP-12 questionnaire	Going to sports or social clubs improves the quality of life for retirees over time.	
Rakhshani et al. 2014 [15] (Iran)	CS	500 60 Y and above 54% M	Health-promoting Profile Health-promoting Lifestyle Profile (HPLP II)	Quality of life (Short Form Health Survey questionnaire (SF-36))	There was a positive relationship between Health-promoting Lifestyle and quality of life in retired older adults.	Physical activity was one of the important predictors of quality of life in retired older adults.
Read et al. 2013 [40] (Australia)	CS	123 80 42% M	Meaningful leisure activity The meaningful leisure activities questionnaire (MLAQ)	Quality of life (Australian WHOQoL-BREF)	There was a positive and significant relationship between significant leisure and quality of life in retirees.	
Reddick & stewart 1994 [48] (USA)	CS	618 73 100% F	Leisure activity participation assessed by responses to four questions	Life satisfaction 18-item Life Satisfaction Index Z (LSIZ)	The strongest predictor of life satisfaction in retirees was leisure activity participation and leisure repertoire planning	
Russell 1987 [51] (India)	CS	210 - 37% M	Recreation activity Modified version of McKechnie’s (1975) Leisure Activities Blank (LAB).	Life satisfaction Shortened version of Neulinger’s (1986) questionnaire, “A Self-Exploration: What Am I Doing: (WAID)	There was no positive correlation between the frequency of participation in recreational activities and life satisfaction in retirement.	Satisfaction with recreation activities was positively related to life satisfaction in retirement.
Sener et al. 2007 [27] (Turkey)	CS	231 65.73 100% M	Leisure activities ‘Leisure Activities Index’	Life Satisfaction Life Satisfaction Index (LSI)	The strongest predictor of life satisfaction in retirees was frequency of participation in leisure activities.	
Walsh et al. 2019 [9] (USA)	CS	373 71 51% M	Sport participation Leisure Attitude Scale	Well-being retirement adjustment and retirement satisfaction scales	This study demonstrated the ongoing value of sports participation in retirement to increase adjustment and satisfaction.	

*CS *Cross-sectional, *L* Longitudinal, *Y*  Year, *M* Male, *F* Female

### The main variables examined in studies

From among the examined papers regarding physical activity dimension, 15 studies had dealt with leisure or participation in leisure, 6 studies physical activity or physical exercise, two studies recreation or participation in recreation, two studies participation in sports, and one study had investigated the profile of health promotion (including physical activity) with regards to the adjustment-associated variables among the retirees. Out of the variables associated with the retirees’ adjustment, 11 studies had dealt with life satisfaction, 5 with wellbeing, 6 with quality of life, 1 with adjustment, and 3 with mental health of retirees in association with physical activity and leisure activities.

### Relationship between variables of physical activity and psychological adjustment variables among the retirees

The relationship between physical activity and psychological adjustment variables among the retirees is summarized in Tables 3 and 4. From among all studies, 11 had dealt with examining the retirees’ life satisfaction; in seven studies, the relationship between leisure activity and life satisfaction was positive [23, 24, 41, 43, 46–48], while in two studies there was no relationship between these variables [49, 50]. Furthermore, two studies had explored participation in leisure with regards to the retirees’ life satisfaction; in one of the studies, there was no relationship [51], while in another study in American sample, the relationship was positive, but again in Indian retiree’s subjects, no correlation was observed between variables [39].

**Table 4:** Frequency of physical activity and its relationship with retirement adjustment

Authors and Year	Relation studied	Relationship PA with RA	Frequency of physical activity	Gender differences in physical activity
Bailey & McLaren 2005 [7]	Physical activity & mental health (Depression)	-	The sample spent more time engaging in physical activity alone than they did with others	NR
Bevil et al. 1994 [46]	Leisure activity & life satisfaction	+	Retirees were engaged in a wide range of leisure activities	No Difference
Brien 1981 [50]	Leisure activities & life satisfaction	NRD	NR	Male
Conde-Sala et al. 2017 [44]	Physical exercise & quality of life	+	44.1% of the sample participated in physical exercise and 79.6% in individual or social leisure activities.	NR
Earl et al. 2015 [14]	Leisure activities & Psychological wellbeing	Exercises with negative affect (+) social activities with positive affect (+)	Home entertainment was the leisure activity that retirees dedicated most time to (27.47 h/w), while vigorous exercises was engaged in rarely (1.84 h/w).	NR
Fly et al. 1981 [54]	Leisure activity & Adjustment	+	NR	No Difference
Fry & ghosh 1980 [39]	Recreation & life satisfaction	+ (USA) NRD (India)	NR	NR
Henning et al. 2021 [26]	Leisure activity & mental health (Depression)	-	Engagement in intellectual, social or physical leisure activities increased after retirement	NR
Iwatsubo et al. 1996 [41]	Leisure activities & life satisfaction	+	NR	NR
Krahe 2011[49]	Leisure participation & life satisfaction	NRD	The most frequently leisure activity in retirees was passive leisure activities such as watching TV or videos.	Passive leisure: male Social leisure: female Active leisure: no difference
Kuvaja-Köllner et al. 2013 [8]	Physical exercise & quality of life	+	Retirement increased the time spent on moderate-to-heavy physical exercise by more than 1 h/w. (mean: 3.30 h/w)	Male
Lee & Hung 2011 [20]	Physical exercise & well-being	+ (frequency) - (intensity)	Retired older adults spent an average of 11.05 h/w in exercise, approximately 1 h and 34.7 m/d.	Stretching exercises: female Walking and hiking: male
Morgan et al. 1991 [53]	Physical activity & psychological well-being	W	NR	Female
Nimrod & Adoni 2006 [24]	Leisure activities & life satisfaction	+	NR	Female
Nimrod & Shrira 2016 [45]	Leisure activities & quality of life	+	NR	NR
Nimrod 2007 [23]	Leisure activities & life satisfaction	+	NR	NR
Nimrod et al. 2008 [52]	Leisure participation & Subjective well-being	+ (Israel) NRD (USA)	The frequency of types of leisure activities after retirement was further increased among Israeli sample than American sample.	Male
Oakley & Pratt 1997 [47]	Leisure activity & life satisfaction	+	NR	NR
Olds et al. 2018 [3]	Physical activity & mental health (depression, stress)	-	Increases in physical activity levels across retirement were modest, LPA (+ 14 m/d), MVPA (+ 4 m/d)	No Difference
Potocnik & Sonnentag 2013 [42]	Participants in sport & quality of life	+	Participation in sports among retirees was 18.8%.	NR
Rakhshani et al. 2014 [15]	Health-promoting Profile (Physical activity) & quality of life	+	Physical activity had the lowest score in the HPL subscales of the retired older adults.	Male
Read et al. 2013 [40]	Leisure activity & quality of life	+	NR	NR
Reddick & stewart 1994 [48]	Leisure activity participation & Life satisfaction	+	NR	The study was performed only on female
Russell 1987 [51]	recreation activity & Life satisfaction	NRD	Retirees seldom participated in the recreation activities (such as sports)	NR
Sener et al. 2007 [43]	Leisure activities & life satisfaction	+	Activities that Quite often done were: audiovisual and reading Activities that hardly ever done were: hobbies, sport and exercise.	The study was performed only on male
Walsh et al. 2019 [9]	Sport participation – wellbeing	+	NR	NR

*PA *Physical Activity, *RA* Retirement Adjustment, *NR* Not reported, *NRD *Not related, *W W*eak, *h/w* Hours per week, *m/d* Min every day, *MVPA* Moderate-to-Vigorous Physical Activity, *LPA* Light Physical Activity

Five studies involved examination of the retirees’ wellbeing; two studies had examined the relationship between leisure activity, two exercise and physical activity, and one participation in sports in association with the retirees’ wellbeing. In exploring the relationship between leisure and wellbeing, one study showed that there was a positive relationship between light exercise and negative affect, as well as between social activity and positive affect [14]. In another study in an Israeli sample, a positive relationship was found, but in the American sample, no relationship was found between leisure and wellbeing [52]. In inspecting the relationship between physical activity and wellbeing, in one study in terms of frequency, there was a positive relationship but regarding intensity, there was a negative relationship between physical activity and wellbeing among the retirees [20]. However, in another study, the relationship between variables was weak and gender associated (weak relationship only in women) [53]. The relationship between participation in physical activity and wellbeing was positive in the study by Walsh et al. [9].

The retirees’ quality of life had been investigated in six studies; in three of them the relationship between exercise, physical activity, and quality of life was positive [8, 15, 44]. Also in two studies, a positive relationship was found between leisure activity and quality of life [40, 45]. Further, in another study a positive relationship was found between participation in physical activity and quality of life of the retirees [42].

From three studies that had dealt with investigating the mental health of retirees, in two studies, there was a negative relationship between physical activity and depression [3, 7]; in another study again a negative relationship was found between leisure and depression of the retirees [26].

Eventually, in a study that had dealt with adjustment of the retirees, there was a positive correlation between leisure activity and adjustment among the retirees [54].

### Frequency, intensity, and type of physical activity, leisure, and associated variables among the retirees

Table 4 reports the extent and frequency of physical activity and leisure activities. As seen in Table 4, out of 26 studies, in 12 of them, the extent and frequency of physical activity has not been reported. Five studies indicated that retirees were mostly involved in leisure activities such as home entertainment, reading, watching TV and movies, as well as other audiovisual activities, while being less engaged in physical activity, exercise, and the like [14, 15, 43, 49, 51]. Specifically, a study indicated that in terms of time allocated to participation in leisure, 60% of the time would be spent on passive leisure activities, 25% on active leisure activities, and 15% on social leisure activities [49]. Regarding the frequency of participation, Henning et al. [26] showed that participation in intellectual, social, or physical activities increased after retirement. In the study by Conde-Sala et al. [44], 44.1% of samples participated in physical exercises and 79.6% in individual or social leisure activities. The prevalence of retirees’ participation in sports club attendance in the study by Potocink & Sonnentag [42] was 18.8%. Considering extent, a study showed that the retirees participated in a wide range of leisure activities [46]. With regards to individual or group participation, a study showed that the retirees would spend more time on participation in individual physical activity [7]. Considering intercultural variables, a study revealed that the frequency of different types of leisure activities after retirement increased more in the Israeli sample than in the American sample [52]. Concerning the time allocated, a study found that retirement increased the time allocated to moderate-to-heavy physical exercise by more than 1 h per week [8]. The results of the study by Olds et al. [3] indicated that elevation of the physical activity level in retirees was average; light exercise had increased by 14 min per day and moderate-to-heavy exercise by 4 min per day. Another study indicated that the retirees would exercise 11 h per week on average, around 1 h and 34 min per day [20].

### Gender differences in the frequency of physical activity and leisure in retirees

Gender differences between men and women in terms of participation in leisure and physical activities are mentioned in Table 4. Among the studies examined in the present systematic review, 24 study had considered both genders, one study only retired women [48], and one study only retired men [43]. In 13 studies, no finding had been reported on the extent and frequency of physical activity and leisure among men and women. Three studies found no difference between men and women regarding participation in leisure and physical activity [3, 46, 54]. In four studies, men had higher involvement than women in leisure and physical activities [8, 15, 50, 52]. A study indicated that although gender comparison was not possible, women had greater participation in physical activities [53]. Also, in a study, the partaking of women in leisure activities was greater with a very slight difference [24]. Further, in a study examining the men and women differences in three types of leisure activities (active, passive, and social), the results showed that women had more contribution in social leisure, while men had greater attending in passive leisure activities. However, there was no difference in terms of participation in active leisure activities [49]. Finally, in a study, women were more active in stretching exercise, while men were more active in walking [20].

## Discussion

This systematic review aimed to investigate the nature of changes in physical activity and leisure activities, as well as the relationship between physical activity, leisure, and psychological adjustment among retirees. The main findings of this systematic review which included 26 studies indicated that there has been a significant and positive correlation between physical activity, leisure, and psychological adjustment among retirees; doing physical activity and enhancing participation in leisure activities were associated with higher adjustment, wellbeing, life satisfaction, quality of life, and mental health. Thus, logically it can be postulated that if people allocated the time for working at pre-retirement to physical activity and participation in active leisure post-retirement, they may experience positive changes in indicators of mental health and psychological adjustment during the retirement period [3]. Having reviewed the studies, we found that physical activity and participation in active leisure played a more important role in psychological adjustment and mental health of retirees compared to passive leisure and doing sedentary activities such as watching TV, reading, and other audiovisual activities.

In a limited number of studies, there was no relationship between the main variables. In the study by Krahe [49] and O’Brien [50], it was found that leisure activities were not significantly associated with life satisfaction. Also, the results of Russell [51] indicated that the frequency of participation in recreational activities had no significant positive relationship with life satisfaction during the retirement period. The notable point was that in all three studies, leisure activities had been measured by life satisfaction. Most probably, other factors than leisure are involved in the retirees’ life satisfaction, causing the different results among studies. Confirming this explanation, the study by Kuykendall et al. [55] showed that factors other than the leisure activity itself, such as satisfaction with the level of leisure activity, may contribute to the adjustment of retirees.

The findings of this systematic review showed that the frequency, intensity, and type of physical activity and leisure time reported in the studies were variable. In many studies, no findings had been reported on the frequency, intensity, and type of physical activity as well as leisure. A number of studies also reported that retirees were mostly involved in passive leisure activities. Specifically, Krahe [49] showed that 60% of the time allocated to leisure in retirees was occupied by passive leisure activities. A few studies have also dealt with the spectrum of participation in different types of activities, group or individual participation, examining the intercultural variables involved in participation and the time spent for physical activity as well as leisure. Therefore, it was difficult to make a general organized assessment of the total physical activity among retirees due to the variability of the results of the studies. Obtaining such variable results among studies can be due to the use of varying instruments, single-case questions, and researcher-made questionnaires for measuring the variables. Hence, further research is required on the use of valid tools for assessment of physical activity which have objective criteria specifically for old age and retirees to better understand the changes in total physical activity.

Considering the intensity of physical activities, it seems that physical activity with low-to-moderate intensity can have a more important role in retirees’ adjustment. The study by Lee and Hung [20] showed that although physical activity can, in terms of frequency, have a positive association with wellbeing among the retirees, a negative correlation was found between physical activity and wellbeing in terms of intensity. Lee and Hung study emphasized low-to-moderate intensity exercise for improving the mental health of the old age and retirees. In this regard, Lahti [56] also indicated that retirement increases the time spent on moderate-intensity activity (such as jogging), while heavy activity (such as walking) decreases. Hence, mental health professionals should encourage retirees to do low-to-moderate intensity activities as well as leisure activities such as walking so that retirees could have better adjustment to the retirement period [57].

Gender differences regarding participation in leisure and physical activity had not been examined in half of the reviewed studies. However, in exploring the other half, several studies indicated that men would, to some extent, participate more than women in physical activity and leisure [8, 15, 50, 52]. Nevertheless, in two studies, women showed greater participation with a very minor difference [24, 53]. This difference was not evident between these two genders in several studies [3, 46, 54]. Regarding gender differences in participation in various leisure activities, a study [49] showed that women participated more in social leisure activities and men in passive leisure activities, while there were no differences in terms of active leisure. It seems that encouraging both retired men and women to do physical activities as well as active and meaningful leisure should be among the top priorities of healthcare policy makers to enhance the retirees’ adjustment and their mental health.

Another notable finding in the present study was that assessment of physical activity or participation in physical exercise has received little attention. Many studies have dealt with evaluating recreational or leisure activities. Nevertheless, such studies can arise from the nature of the retirement period and old age. Retirement increases free time and reduces work-related stress; however, the increased time is not necessarily allocated to health-promoting activities, rather the extra leisure is spent on unhealthy lifestyle [58]. Meanwhile, retirees may get involved less with physical activity and physical exercises due to various factors. Confirming this finding, it has been shown that shortage of financial resources during the retirement period, preference of sedentary activities, negative experience of participation in physical activities during the young ages, and low awareness of the advantages of physical activity have been mentioned as the key obstacles against participation in physical activity for retirees [13].

Also, another interesting point found in this study was that except for five studies that had used a longitudinal approach for measuring leisure and physical activity, all studies had been done as cross-sectional. Performing longitudinal studies is very essential for measuring the long-term effect of physical activity on retirees’ adjustment, so that one could find whether the effect of exercise and physical activity would be maintained for the long time or it is a short-term phenomenon. Of course, demographic variables, socioeconomic factors and other confounding variables should be evaluated for real measurement of variables.

Based on the analysis obtained from this systematic review, the focus of studies before 2010 was more on leisure activities, but after 2010, although studies on physical activity have increased relatively, it is necessary to focus more on physical activity and active leisure time in future studies. In terms of the number of samples, 17 studies have less than 500 people, three studies have 500 to 1000 people, and six studies have chosen more than 1000 people as samples; therefore, it is highly recommended to select a sample with a high number in future studies. In terms of geographical focus, the study in the countries of the African and South American continents is very limited, and it is necessary to increase the concentration of studies according to the current research objectives in these countries.

Also, the results of the analysis in the current systematic review in terms of the investigated variables showed that out of 26 studies, 15 studies were conducted on leisure activities and their relationship with psychological adjustment variables, the focus on physical activity was very low; Therefore, the situation of retirees in terms of physical activity and the amount of time participation in physical exercises should be investigated more broadly to psychological adjustment variables. Among the variables of psychological adjustment, although the life satisfaction of retirees has been investigated more than others, the focus of all studies has been on the relationship between life satisfaction and leisure time and participation in recreation, and conducting studies on the relationship between life satisfaction and physical activity and Participation in sports has been very limited, so it is necessary to conduct more extensive studies in this field. Also, based on the analysis of the findings of the present systematic review studies, the relationship between the quality of life, mental health, and adjustment variables to leisure time and physical activity has been evaluated positively, but the relationship between the well-being variable and leisure time and physical activity is variable. Therefore, it is recommended to carry out further studies for a more comprehensive examination of the relationship between these variables.

### Strengths and limitations

Since there have been insufficient data about the effect of adjustment on the retirees’ physical activity, thus no causal relationship can be inferred between physical activity and retirees’ adjustment based on the current literature. Nevertheless, the present systematic review for the first time presents evidence on the relationship between physical activity, leisure, and psychological adjustment among the retirees. In the present systematic review, evidence indicated that retirees mostly participated in passive and sedentary leisure activities. This study sets the basis for retirees’ to place more emphasis on low-to-moderate intensity physical activity for enhancing adjustment.

## Conclusion

Eventually, it can be stated that active leisure and physical activities cause enhanced psychological adjustment of retirees. In terms of gender, men had somewhat more participation in leisure and physical activities compared to women. Due to use of various tools and inaccuracies in measuring the studied variables, the frequency and type of physical activity as well as leisure reported in the studies were variable. Therefore, the global organized evaluation of the total physical activity among retirees was difficult. The common challenging issue of reviewed studies in the present systematic review was that most retirees were involved in passive leisure activities, and participation in active leisure and physical activities was relatively low. Thus, emphasis should be placed on low-to-moderate intensity physical activity, which are both risk-free and interesting, in the routine of these people’s lives to improve adjustment and reduce risks of physical damage. Also, future studies can deal with usage of more valid physical activity measurement instruments specially designed for old age and retirement. They can also conduct further longitudinal studies to measure the long-term effects of physical activity on the retirees’ adjustment by controlling the confounding variables. On the other hand, in order to ensure design of interventions for enhancing the retirees’ adjustment, further research should be done on reciprocal relationships between physical activity, leisure, and retirees’ adjustment. Finally, in the selection of samples, a larger number of retirees should be examined, especially in terms of evaluating physical activities and participation in active leisure activities and their relationship with the life satisfaction and well-being of retirees.

## Supplementary Information

**Additional file 1.**

## Data Availability

All data generated or analysed during this study are included in this article (and its supplementary information files).

## Abbreviations

Y: Yes (In Table 1)
U: Unclear
N: NO
CS: Cross-sectional
L: Longitudinal
Y: Year (In Table 3)
M: Male
F: Female
PA: Physical Activity
RA: Retirement Adjustment
NR: Not Reported
NRD: Not Related
W: Weak
H/W: Hours per Week
M/D: Min every Day
MVPA: Moderate-to-Vigorous Physical Activity
LPA: Light Physical Activity

## References

[1] Luborsky MR, LeBlanc IM (2003). Cross-cultural perspectives on the concept of retirement: an analytic redefinition. J Cross-Cult Gerontol.

[2] Kim JE, Moen P (2002). Retirement transitions, gender, and psychological well-being: a life-course, ecological model. The Journals of Gerontology Series B: Psychological Sciences and Social Sciences.

[3] Olds T, Burton NW, Sprod J, Maher C, Ferrar K, Brown WJ (2018). One day you’ll wake up and won’t have to go to work: the impact of changes in time use on mental health following retirement. PLoS ONE.

[4] Kohl HW, Craig CL, Lambert EV, Inoue S, Alkandari JR, Leetongin G (2012). The pandemic of physical inactivity: global action for public health. The lancet.

[5] Lee SEJ, Lobelo F, Puska P, Blair SN, Katzmarzyk PT (2012). Effect of physical inactivity on major non-communicable diseases worldwide: an analysis of burden of disease and life expectancy. The lancet.

[6] An KY. Physical activity level in Korean adults: the Korea National health and nutrition examination survey 2017. Epidemiology and Health. 2019;41:1-10. 10.4178/epih.e2019047 PMC6928466 31801321

[7] Bailey M, McLaren S (2005). Physical activity alone and with others as predictors of sense of belonging and mental health in retirees. Aging Ment Health.

[8] Kuvaja-Köllner V, Valtonen H, Komulainen P, Hassinen M, Rauramaa R (2013). The impact of time cost of physical exercise on health outcomes by older adults: the DR’s EXTRA study. Eur J Health Econ.

[9] Walsh DW, Green BC, Holahan C, Cance JD, Lee D (2019). Healthy aging? An evaluation of sport participation as a resource for older adults in retirement. J Leisure Res.

[10] McMunn A, Nazroo J, Wahrendorf M, Breeze E, Zaninotto P (2009). Participation in socially-productive activities, reciprocity and wellbeing in later life: baseline results in England. Ageing Soc.

[11] Allender S, Hutchinson L, Foster C (2008). Life-change events and participation in physical activity: a systematic review. Health Promot Int.

[12] Brown WJ, Heesch KC, Miller YD (2009). Life events and changing physical activity patterns in women at different life stages. Ann Behav Med.

[13] Barnett I, van Sluijs EM, Ogilvie D (2012). Physical activity and transitioning to retirement: a systematic review. Am J Prev Med.

[14] Earl JK, Gerrans P, Halim VA (2015). Active and adjusted: investigating the contribution of leisure, health and psychosocial factors to retirement adjustment. Leisure Sci.

[15] Rakhshani T, Shojaiezadeh D, Lankarani KB, Rakhshani F, Kaveh MH, Zare N. The association of health-promoting lifestyle with quality of life among the Iranian elderly. Iranian Red Crescent Medical Journal. 2014;16(9):1-6. 10.5812/ircmj.18404 PMC4270660 25593729

[16] Carlson JA, Sallis JF, Conway TL, Saelens BE, Frank LD, Kerr J (2012). Interactions between psychosocial and built environment factors in explaining older adults’ physical activity. Prev Med.

[17] Kouvonen A, De Vogli R, Stafford M, Shipley MJ, Marmot MG, Cox T (2012). Social support and the likelihood of maintaining and improving levels of physical activity: the Whitehall II study. Eur J Public Health.

[18] Shelton RC, McNeill LH, Puleo E, Wolin KY, Emmons KM, Bennett GG (2011). The association between social factors and physical activity among low-income adults living in public housing. Am J Public Health.

[19] Barbosa LM, Monteiro B, Murta SG (2016). Retirement adjustment predictors—A systematic review. Work Aging and Retirement.

[20] Lee Y-J, Hung W-L (2011). The relationship between exercise participation and well-being of the retired elderly. Aging Ment Health.

[21] Van Solinge H, Henkens K (2008). Adjustment to and satisfaction with retirement: two of a kind?. Psychol Aging.

[22] Wang M (2007). Profiling retirees in the retirement transition and adjustment process: examining the longitudinal change patterns of retirees’ psychological well-being. J Appl Psychol.

[23] Nimrod G (2007). Expanding, reducing, concentrating and diffusing: Post retirement leisure behavior and life satisfaction. Leisure Sci.

[24] Nimrod G, Adoni H (2006). Leisure-styles and life satisfaction among recent retirees in Israel. Ageing Soc.

[25] Osborne JW (2012). Psychological effects of the transition to retirement. Can J Counselling Psychother.

[26] Henning G, Stenling A, Bielak AA, Bjälkebring P, Gow AJ, Kivi M (2021). Towards an active and happy retirement? Changes in leisure activity and depressive symptoms during the retirement transition. Aging Ment Health.

[27] Wang M, Henkens K, van Solinge H (2011). Retirement adjustment: a review of theoretical and empirical advancements. Am Psychol.

[28] Orthner DK. Leisure activity patterns and marital satisfaction over the marital career. Journal of Marriage and the Family. 1975;37(1):91–102.

[29] Vansweevelt N, Boen F, van Uffelen J, Seghers J (2022). Socioeconomic differences in physical activity and sedentary Behavior during the Retirement transition: a systematic review of Longitudinal Studies. J Phys Activity Health.

[30] Wilson DM, Palha P (2007). A systematic review of published research articles on health promotion at retirement. J Nurs Scholarsh.

[31] Mänty M, Kouvonen A, Lallukka T, Lahti J, Lahelma E, Rahkonen O (2018). Changes in physical and mental health functioning during retirement transition: a register-linkage follow-up study. Eur J Pub Health.

[32] Li W, Ye X, Zhu D, He P (2021). The longitudinal association between retirement and depression: a systematic review and meta-analysis. Am J Epidemiol.

[33] Odone A, Gianfredi V, Vigezzi G, Amerio A, Ardito C, d’Errico A et al. Does retirement trigger depressive symptoms? A systematic review and meta-analysis. Epidemiology and psychiatric sciences. 2021;30(e77):1-24. 10.1017/S2045796021000627 PMC8679838 35048820

[34] Amorim SM, França LHdFP (2019). Retirement well-being: a systematic review of the literature. Trends in psychology.

[35] Solhi M, Pirouzeh R, Zanjari N, Janani L. Dimensions of Preparation for Aging: A Systematic Review. Medical Journal of the Islamic Republic of Iran. 2022;36(81):1-9. 10.47176/mjiri.36.81 PMC9448489 36128301

[36] Moher D, Shamseer L, Clarke M, Ghersi D, Liberati A, Petticrew M (2015). Preferred reporting items for systematic review and meta-analysis protocols (PRISMA-P) 2015 statement. Syst reviews.

[37] Stang A (2010). Critical evaluation of the Newcastle-Ottawa scale for the assessment of the quality of nonrandomized studies in meta-analyses. Eur J Epidemiol.

[38] Santos WMd, Secoli SR, Puschel VAdA. The Joanna Briggs Institute approach for systematic reviews. Revista latino-americana de enfermagem. 2018;26:1-2. 10.1590/1518-8345.2885.3074 PMC6248737 30462787

[39] Fry PS, Ghosh R (1980). Attributional differences in the life satisfactions of the elderly: a cross-cultural comparison of asian and United States subjects. Int J Psychol.

[40] Read JM, Muller JJ, Waters LE (2013). The importance of latent benefits and meaningful leisure activity in predicting quality of life in australian retirees. Australian J Career Dev.

[41] Iwatsubo Y, Derriennic F, Cassou B, Poitrenaud J (1996). Predictors of life satisfaction amongst retired people in Paris. Int J Epidemiol.

[42] Potočnik K, Sonnentag S (2013). A longitudinal study of well-being in older workers and retirees: the role of engaging in different types of activities. J Occup Organizational Psychol.

[43] Şener A, Terzioğlu R, Karabulut E (2007). Life satisfaction and leisure activities during men’s retirement: a turkish sample. Aging and Mental Health.

[44] Conde-Sala JL, Portellano-Ortiz C, Calvó-Perxas L, Garre-Olmo J (2017). Quality of life in people aged 65 + in Europe: associated factors and models of social welfare—analysis of data from the SHARE project (Wave 5). Qual Life Res.

[45] Nimrod G, Shrira A (2016). The paradox of leisure in later life. Journals of Gerontology Series B: Psychological Sciences and Social Sciences.

[46] Bevil CA, O’Connor PC, Mattoon PM (1994). Leisure activity, life satisfaction, and perceived health status in older adults. Gerontol Geriatr Educ.

[47] Oakley C, Pratt J (1997). Voluntary work in the lives of post-retirement adults. Br J Occup Therapy.

[48] Riddick CC, Stewart DG (1994). An examination of the life satisfaction and importance of leisure in the lives of older female retirees: a comparison of blacks to whites. J Leisure Res.

[49] Krahe LM (2011). Leisure participation and the life, health, leisure and retirement satisfaction of retirees: a case study of Port Macquarie, Australia. Int J Disabil Hum Dev.

[50] O’Brien GE (1981). Leisure attributes and retirement satisfaction. J Appl Psychol.

[51] Russell RV (1987). The importance of recreation satisfaction and activity participation to the life satisfaction of age-segregated retirees. J Leisure Res.

[52] Nimrod G, Janke MC, Kleiber DA (2008). Retirement, activity, and subjective well-being in Israel and the Unites States. World Leisure Journal.

[53] Morgan K, Dalleosso H, Bassey EJ, Ebrahim S, Fentem P, Aire T (1991). Customary physical activity, psychological well-being and successful ageing. Ageing Soc.

[54] Fly JW, Reinhart GR, Hamby R (1981). Leisure activity and adjustment in retirement. Sociol Spectr.

[55] Kuykendall L, Tay L, Ng V (2015). Leisure engagement and subjective well-being: a meta-analysis. Psychol Bull.

[56] Lahti J, Laaksonen M, Lahelma E, Rahkonen O (2011). Changes in leisure-time physical activity after transition to retirement: a follow-up study. Int J Behav Nutr Phys Activity.

[57] Windle G (2014). Exercise, physical activity and mental well-being in later life. Reviews in Clinical Gerontology.

[58] Feng J, Li Q, Smith JP (2020). Retirement effect on health status and health behaviors in urban China. World Dev.

