# Supplementary material for: Physical activity and psychological adjustment among retirees: a systematic review
Source: BMC Public Health. 2023 Jan 28;23:194. doi: 10.1186/s12889-023-15080-5 (PMC9884422; doi:10.1186/s12889-023-15080-5)
Supplement: Supplementary file 1 — Additional file 1. [file 12889_2023_15080_MOESM1_ESM.docx]

PubMed: Title/Abstract: 20 February 2022

| search | query |  |
| --- | --- | --- |
| #1 | **(((((("Physical Activ*"[Title/Abstract]) OR (Exercise[Title/Abstract])) OR (Sport[Title/Abstract])) OR (recreation[Title/Abstract])) OR ("leisure activ*"[Title/Abstract])) OR ("Physical exercise"[Title/Abstract])) OR ("motor activ*"[Title/Abstract])** | **451402** |
| #2 | **(((((Adjustment[Title/Abstract]) OR (Well-Being[Title/Abstract])) OR ("Quality Of Life"[Title/Abstract])) OR (Mental health[MeSH])) OR ("Mental health"[Title/Abstract]) OR (Satisfaction[Title/Abstract]))** | **868568** |
| #3 | 1 AND 2 | 50508 |
| #4 | **(((Retirement[Title/Abstract]) OR (Retirement[MeSH])) OR (retir*[Title/Abstract]))** | **27995** |
| #5 | 3 AND 4 | 421 |
|  | Only original articles | 383 |

ISI Web of Science: Title: 20 February 2022

| search | query |  |
| --- | --- | --- |
| #1 | **((((((TI=("Physical Activ*")) OR TI=(Exercise)) OR TI=(Sport)) OR TI=(recreation)) OR TI=("leisure activ*")) OR TI=("Physical exercise")) OR TI=("motor activ*")** | **331828** |
| #2 | **((((TI=(Adjustment)) OR TI=(Well-Being)) OR TI=("Quality Of Life")) OR TI=("Mental health")) OR TI=(Satisfaction)** | **350584** |
| #3 | **#1 AND #2** | **7034** |
| #4 | **((((TI=(Retirement)) OR TI=(retiring)) OR TI=(retire)) OR TI=(Retirees)) OR TI=(Retired)** | **15894** |
| #5 | **#3 AND #4** | **18** |
|  | **Only original articles** | **16** |

ISI Web of Science: Abstract: 20 February 2022

| search | query |  |
| --- | --- | --- |
| #1 | **((((((AB=("Physical Activ*")) OR AB=(Exercise)) OR AB=(Sport)) OR AB=(recreation)) OR AB=("leisure activ*")) OR AB=("Physical exercise")) OR AB=("motor activ*")** | 603086 |
| #2 | **((((AB=(Adjustment)) OR AB=(Well-Being)) OR AB=("Quality Of Life")) OR AB=("Mental health")) OR AB=(Satisfaction)** | 1084394 |
| #3 | **#1 AND #2** | 61467 |
| #4 | **((((AB=(Retirement)) OR AB=(retiring)) OR AB=(retire)) OR AB=(Retirees)) OR AB=(Retired)** | 33114 |
| #5 | **#3 AND #4** | 506 |
|  | **Only original articles** | 465 |

Scopus: **Title/Abstract:** 21 February 2022

| search | query |  |
| --- | --- | --- |
| 1 | TITLE-ABS ( "Physical Activ*"  OR  exercise  OR  sport  OR  recreation  OR  "leisure activ*"  OR  "Physical exercise"  OR  "motor activ*" ) | 858614 |
| 2 | TITLE-ABS ( adjustment  OR  well-being  OR  "Quality Of Life"  OR  "Mental health"  OR  satisfaction ) | 1504453 |
| 3 | **#1 AND #2** | 74746 |
| 4 | TITLE-ABS ( retirement  OR  retiring  OR  retire  OR  retirees  OR  retired ) | 57241 |
| 5 | **#3 AND #4** | 683 |
|  | **Only original articles** | 594 |
